# Social learning for resilient data fusion against data falsification attacks

**DOI:** 10.1186/s40649-018-0057-7

**Published:** 2018-10-25

**Authors:** Fernando Rosas, Kwang-Cheng Chen, Deniz Gündüz

**Affiliations:** 1 Centre of Complexity Science and Department of Mathematics, Imperial College London, Kensington, London, SW72AZ UK; 2 Department of Electrical and Electronic Engineering, Imperial College London, Kensington, London, SW72AZ UK; 3 Department of Electrical Engineering, University of South Florida, 4202 E Fowler Ave, Tampa, FL 33620 USA

**Keywords:** Distributed decision-making, Data fusion, Sensor networks, Social networks, Data falsification attacks, Byzantine nodes, Collective behaviour, Multi-agent systems, Social learning, Information cascades

## Abstract

**Background:**

Internet of Things (IoT) suffers from vulnerable sensor nodes, which are likely to endure data falsification attacks following physical or cyber capture. Moreover, centralized decision-making and data fusion turn decision points into single points of failure, which are likely to be exploited by smart attackers.

**Methods:**

To tackle this serious security threat, we propose a novel scheme for enabling distributed decision-making and data aggregation through the whole network. Sensor nodes in our scheme act following social learning principles, resembling agents within a social network.

**Results:**

We analytically examine under which conditions local actions of individual agents can propagate through the network, clarifying the effect of Byzantine nodes that inject false information. Moreover, we show how our proposed algorithm can guarantee high network performance, even for cases when a significant portion of the nodes have been compromised by an adversary.

**Conclusions:**

Our results suggest that social learning principles are well suited for designing robust IoT sensor networks and enabling resilience against data falsification attacks.

## Background

### Motivation

Internet of Things (IoT) is expected to play a central role in future digital society. However, to fully adopt this technology, it is crucial to guarantee its security, specially for public utilities whose safety is essential for the well-being of our society [1]. Recent cyber-attacks that created significant damage have been widely reported, e.g. the self-propagating malware *WannaCry* that caused a infamous worldwide network hack in May 2017 [2]. Developing technologies that can guarantee the safety of large information networks, such as IoT, is a challenging but urgent need. As information networks get more closely intertwined within our daily lives, ensuring their security and thus safety is becoming an even more challenging issue.

As the level of security is typically determined by the weakest link, a major dilemma of IoT security lies in the low-complexity sensor networks that are located at the network edge. These sensor networks are usually composed by a large number of autonomous electronic devices, which collect critical information for the control and operation of IoT [3, 4]. By monitoring extensive geographical areas, these networks can enable a wide range of services to society, becoming a key element for the well-being of future smart cities [5, 6]. These networks may also perform sensitive tasks, including the surveillance over military or secure zones, intrusion detection to private property, monitoring of drinkable water tanks and protection from chemical attacks [7, 8].

Although the design of secure wireless sensor networks have been widely studied (e.g. [9–11] and references therein), there remain many open problems of both theoretical and engineering nature [12]. In particular, as the number of sensors is usually very large, precise management of them is challenging or even infeasible. A significant portion of the sensors might be deployed in unprotected areas, where it is impossible to ensure their physical or cyber security (e.g. war zones, or regions easily accessed by adversaries). Furthermore, sensor nodes are generally not tamper-proof due to cost restrictions, and have limited computing and networking capabilities. Therefore, they may not be capable of employing complex cryptographic or security protocols.

The vulnerability of sensor nodes makes them potential victims of cyber/physical attacks driven by intelligent adversaries. Attacks to information networks are usually categorized into *outsider attacks* and *insider attacks*. Outsider attacks include (distributed) denial of service (DoS) attacks, which use the broadcasting nature for wireless communications to disrupt the communications capabilities [10]. In contrast, in insider attacks the adversary “recruits” sensor nodes by malware through cyber/wireless means, or directly by physical substitution [13]. Following the classical *Byzantine generals problem* [14], these “Byzantine nodes” are authenticated, and recognized as valid members of the network. Byzantine nodes can hence generate false data, exhibit arbitrary behaviour, and collude with others to create network malfunctions. In general, insider attacks are considered to be more potentially harmful to information networks than outside attacks.

The effect of Byzantine nodes and data falsification over distributed sensor networks has been intensely studied; the impact over the network performance has been characterized, and various defense mechanisms has been proposed (c.f. [15] for an overview, and also [16–20] for some recent contributions). However, all these works focus on networks with star or tree topology, and rely on centralizing the decision-making in special nodes, called “fusion centers” (FCs), which gather all the sensed data. Therefore, a key element in these approaches is a strong division of labour: ordinary sensor nodes merely sense and forward data, while the processing is done exclusively at the FC corresponding to a *distributed-sensing/centralized-processing* approach. This literature implicitly assume that the FCs are capable of executing secure coding and protocols, and hence, are out of the reach of attackers. However, large information networks might require another kind of mediator devices, known as data aggregators (DAs), which have the capability to access the cloud through high-bandwidth communication links [21]. DAs are attractive targets for insider attacks, as they might also be located in unsafe locations due to the limited range of sensor node radios. Please note that a tampered DA can completely disable the sensing capabilities of all the nodes whose information has been aggregated, generating a single point of failure that is likely to be exploited by smart adversaries [22].

An attractive route to address this issue is to consider *distributed-sensing/distributed-processing* schemes, which avoid centralized decision-making by distributing processing tasks throughout the network [23]. However, the design of practical distributed-sensing/distributed-processing schemes is a challenging task, as collective computation phenomena usually exhibit highly non-trivial features [24, 25]. In effect, even though the distributed-sensing literature is vast (for classic references c.f. [26–28], and more modern surveys see [3, 4, 29, 30]), the construction of optimal distributed schemes is in general NP-hard [31]. Moreover, although in many scenarios the optimal schemes can be characterized as a set of thresholds for likelihood functions, the determination of these thresholds is usually an intractable problem [26]. For example, homogeneous thresholds can be suboptimal even for networks with similar sensors arranged in star topology [32], being only asymptotically optimal in the network size [33]. Moreover, symmetric strategies are not suitable for more complicated network topologies, requiring heuristic methods.

### Distributed decision-making and social learning

In parallel, significant research efforts have been dedicated to analysing *social learning*, which refers to the decision-making processes that take place within social networks [34]. In these scenarios, agents make decisions based on two elements: private information that represents agent’s personal knowledge, and social information derived from previous decisions made by the agent’s peers [35].

Social learning has been investigated in pioneering works that study sequential decision-making of Bayesian agents over simple social network structures [36, 37]. These models showed how, thanks to social interactions, individuals with weak private signals can harvest information from the decisions of other agents [38]. Interestingly, it was also found that aggregation of rational decisions through *information cascades* could generate suboptimal collective responses, degrading the “wisdom of the crowds” into mere herd behaviour. After these initial findings, researchers have aimed at developing a deeper understanding of information cascades extending the original models by considering more general cost metrics [39–41], and by studying the effects of the network topology on the aggregated behaviour [42–45]. Non-Bayesian learning models have also been explored, where agents use simple rule-of-thumb methods to exchange information [46–52].

Social learning plays a crucial role in many important social phenomena, e.g. in the adoption or rejection of new technology, or in the formation of political opinions [34]. Social learning models are particularly interesting for studying information cascades and herd dynamics, which arises when the social information pushes all the subsequent agents to ignore their own personal knowledge and adopt a homogeneous behaviour [37]. Moreover, there have been a renewed interest in understanding information cascades in the context of e-commerce and digital society [45]. For example, information cascades might have tremendous consequences in online stores where customers can see the opinions of previous customers before deciding to buy a product, or in the emergence of viral media contents based on sequential actions of “like” or “dislike”. Therefore, developing a deep understanding of the mechanics behind information cascades, and how they impact social learning, is fundamental for our modern networked society.

The main motivation behind this article is to explore the connections between social learning and secure sensor networks, building a bridge between the research done separately by economists and sociologist on one side and electrical engineers and computer scientists on the other. A key insight for establishing this connection is to realize that each agent’s decision corresponds to a compressed description of his/her private information. Therefore, the fact that agents cannot access the private information of others, but can only observe their decisions, can be understood as a constraint on the communication resources. In this way, social learning can be regarded as an information network that performs distributed inference under communication constraints (see Table 1). Moreover, it would be natural to use social learning principles in the design of distributed-sensing/distributed-processing schemes, with the hope that this might enable additional robustness to decision-making processes in sensor networks.

**Table 1:** Table of correspondances between distributed detection in sensor networks and social learning in social networks

Distributed detection	Social learning
Sensor node	Social agent
Communication range	Social neighbourhood
Environmental variables	State of the world
Noisy measurement	Private information
Local processing	Agent’s decision
Bandwidth constraints	Decision sharing

### Contributions

In contrast to almost all the existing research, this work considers powerful topology-aware data falsification attacks, where the adversary knows the network topology and leverages this knowledge to take control of the most critical nodes of the network—either regular nodes, DAs or FCs. This represents a worst-case scenario where the network structure has been disclosed or inferred through network tomography via traffic analysis [53]. The reason why this adversary model has not been popular in the literature might be because traditional distributed-sensing schemes do not offer any resistance against this kind of attack.

This works presents a distributed-sensing/distributed-processing scheme for sensor networks that uses social learning principles in order to deal with a topology-aware adversary. The scheme is a threshold-based data fusion strategy, related to those considered in [26]. However, its relationship with social decision-making allows an intuitive understanding of its mechanisms. For avoiding security threats introduced by FCs, our scheme adopt tandem or serial decision sequencing [27, 54–57]. It is noted that, contrasting with some related literature, our analysis does not focus on optimality aspects of data fusion, but aims to illustrate how distributed decision-making can enable network resilience against powerful topology-aware data falsification attacks. We demonstrate how network resilience hold even when a significant number of nodes have been compromised.

Our work exploits a positive effect of information cascades that have been overlooked before: information cascades make a large number of agents/nodes to hold equally qualified estimators, generating many locations where a network operator can collect aggregated data. Therefore, information cascades are crucial in our solution for avoiding single points of failure. For enabling a better understanding of information cascades, this work extends results presented in [58] providing a mathematical characterization of information cascades under data falsification attacks. In particular, our results clarify the conditions upon which local actions of individual agents can propagate across the network, compromising the collective performance. These results provide a first step towards the clarification of these non-trivial social dynamics, enriching our understanding of decision-making processes in biased social networks.

This paper expands the ideas presented in [59] by developing a formalism that allows considering incomplete or imperfect social information. This formalism is used to overcome the strongest limitation of the scheme presented in [59], namely the fact that each node was required to overhear and store all the previous transmissions in the network. Clearly this cannot take place in a large sensor network, due both to the storage constraints of the nodes, and to the large energy consumption required to transmit and receive across all pairs of nodes [60]. Therefore, this research presents an important step towards practical applications.

The rest of this article is structured as follows: “System model and problem statement” section introduces the system model, describing the network controller and the adversary behaviour. Our social learning data fusion scheme is then described in “Social learning as a data aggregation scheme” section, where some basic statistical properties are explored, and a practical algorithm for implementing the decision rule is derived. “Information cascade” section analyses the mathematical properties of the decision process, providing a geometrical description and a characterization of information cascades. All these ideas are then illustrated in a concrete scenario in “Proof of concept” section. Finally, “Conclusions” section summarizes our main conclusions.

Notation: uppercase letters are used to denote random variables, i.e. *X*, and lowercase letters their realizations, e.g. *x*. Boldface letters $\varvec{X}$ and $\varvec{x}$ represent random vectors and their realizations, respectively. Also, $\mathbb {P}_{w}\left\{ X=x|Y=y \right\} = \mathbb {P}\left\{ X=x|Y=y,W=w \right\}$ is used as a shorthand notation. A table summarizing the symbols and notation used through this article can be found in Appendix D.

## System model and problem statement

### System model

We consider a sensor network of *N* nodes, each corresponding to an information-processing device that has been deployed in an area of interest. Each node is equipped with sensory equipment to track variables of interest following a scheduled duty cycle. The measurement of the *n*-th sensor node is denoted by $S_n,$ taking values over a set $\mathcal {S} \subset \mathbb {R}$ that can be discrete or continuous.^1^ Based on these signals, the network needs to infer the value of an underlying binary variable *W*.

We consider networks where all the nodes have equal sensing capabilities, that is, the signals $S_n$ are assumed to be identically distributed. Unfortunately, the general distributed detection problem for arbitrarily correlated signals is known to be NP-hard [31]. Hence, for the sake of tractability, it is assumed that the variables $S_1,\dots , S_N$ are conditionally independent given the event $\{W=w\},$ 2 following a probability distribution denoted by $\mu _w.$ It is also assumed that both $\mu _0$ and $\mu _1$ are absolutely continuous with respect to each other [67], i.e. no particular signal determines *W* unequivocally. This property guarantees that the log-likelihood ratio of these two distributions is always well defined, being given by the logarithm of the corresponding Radon–Nikodym derivative^3^ $\Lambda _S(s) = \log \frac{d \mu _1}{d \mu _0} (s) .$

In addition to sensing hardware, each node is equipped with limited computing capability and a radio to wirelessly transit and receive data. Two nodes in the network are assumed to be connected if they can exchange information wirelessly. Note that, sensor nodes usually have a very limited battery budget, which imposes severe restrictions on their communication capabilities [68]. Therefore, it is assumed that each node forwards its data to others only by broadcasting a binary variable $X_n.$ These simple signals do not impose an additional burden on the communication resources, as they could be appended to existent wireless control packages and viceversa, or could be shared by light, ultrasound or other alternative media.

We focus on the case in which the sensing capabilities of each sensor are limited, and hence, any inference about *W* made based only on the sensed data $S_n$ cannot achieve a high accuracy. Interestingly, due to the nature of wireless broadcasting, nearby transmissions can be overheard and their information can be fused with what is extracted from the local sensor. The information that a node can extract from overhearing transmissions of other nodes is called “social information”, contrasting with the “sensorial information” that is obtained from the sensed signal $S_n.$

Without loss of generality, nodes transmit their signals sequentially according to their indices (i.e. node 1 transmits first, then node 2, etc.).^4^ It is assumed that this sequence is randomly chosen, and can be changed by the network operator at any time and be re-distributed through the network (c.f. “The sensor network operator and the adversary” section). In general the broadcasted signals $X_1,\dots ,X_{n-1}$ might not be directly observable by the *n*-th agent because of various restrictions, including range limitations of the node’s receiver radio [70], or the limited duty cycles imposed by battery restrictions [68]. Therefore, the social observations obtained by the *n*-th node are represented by $\varvec{G}_n\in \mathcal {G}_n,$ which can be a random scalar, vector, matrix or other mathematical object. Some cases of interest are as follows:

(i) The *k* previous decisions: $\varvec{G}_n = (X_{n-k},\dots ,X_{n-1}).$
(ii) The average value of all the previous decisions: $\varvec{G}_n=\frac{1}{n-1} \sum _{k=1}^{n-1} X_k.$
(iii) The decisions of agents connected by an Erdös–Rényi random network with parameter $\xi \in [0,1],$ i.e. $\varvec{G}_n=(Z_1,\dots ,Z_{n-1}) \in \{0,1,e\}^{n-1},$ where
  1 $$\begin{aligned} Z_k = {\left\{ \begin{array}{ll} X_k \quad & \text {with probability }\xi , \\ e \quad & \text {with probability } 1-\xi .\end{array}\right. } \end{aligned}$$

Please note that the Erdös–Rényi model in (iii) has only been used as an illustrative example, and it can be easily generalized to consider the topology of any stochastic network of interest.

In this work, we study the social dynamics based on the properties of the transition probability from state $\varvec{g'}\in \mathcal {G}_{n-1}$ to $\varvec{g}\in \mathcal {G}_{n},$ as given by the conditional probabilities

2 $$\begin{aligned} \beta _w^n(\varvec{g} | x_{n-1},\varvec{g'}) := \mathbb {P}_{w}\left\{ \varvec{G}_n= \varvec{g} | X_{n-1}=x_{n-1}, \varvec{G}_{n-1}= \varvec{g'} \right\} , \end{aligned}$$

where $x_{n-1}\in \{0,1\}.$ It is also assumed that the social dynamics are causal, meaning that $\varvec{G}_n$ is conditionally independent of $S_m$ given *W* for all $m\ge n.$

### The sensor network operator and the adversary

The network is managed by a network operator, who is an external agent that uses the network as a tool to build an estimate of *W*. The network operator is opposed by an adversary, whose goal is to disrupt the inference capabilities of the network. For this aim, the adversary controls a group of authenticated Byzantine nodes without being noticed by the network operator, which have been captured by malware through cyber/wireless means, or by physical substitution.

The overall performance of a network of *N* nodes is defined by the accuracy of the inference of the last node in the decision sequence. As the decision sequence is generated randomly by the network operator, every node is equally likely to be at the end of the decision sequence. It is further assumed that the adversary has no knowledge of the decision sequence, as it can be chosen at run-time and changed regularly. Therefore, as the adversary has no reason to target any particular node in the network, hence, it is reasonable to assume that the adversary captures nodes randomly. Byzantine nodes are, hence, assumed to be uniformly distributed over the network.

For simplicity, we model the strength of the attack with a single parameter $p_{\text{b}},$ which corresponds to the probability of a node being compromised.^5^ Moreover, we assume that the capture probability does not depend on *W*. Hence, the number of Byzantine nodes, denoted by $N^*,$ is a Binomial random variable with $\mathbb {E} \left\{ N^* \right\} = p_{\text{b}}N.$ Due to the law of large numbers, $N^*\approx p_{\text{b}}N$ for a large network, and hence, $p_{\text{b}}$ is also the ratio of expected Byzantine nodes in the network, which is the traditional metric for attack strength used in the literature.

For enabling data processing and forwarding, the network operator defines a *strategy*, i.e. a data fusion scheme given by a collection of (possibly stochastic) functions $\{\pi _n\}_{n=1}^\infty,$ such that $\pi _n:\mathcal {S}\times \mathcal {G}_n \rightarrow \{0,1\}$ for all $n\in \mathbb {N}.$ On the other hand, the adversary can freely set the values of the binary signals transmitted by Byzantine nodes. This can be modelled as a random mapping $C{:}\, \{0,1\}\rightarrow \{0,1\}$ that corrupts broadcasted signals. Therefore, the signal broadcasted by the *n*-th node is given by

3 $$\begin{aligned} X_n = {\left\{ \begin{array}{ll} C(\pi _n(S_n,\varvec{G}_n)) \quad & \text {with probability }p_{\text{b}},\; \text {and} \\ \pi _n(S_n,\varvec{G}_n) \quad & \text {otherwise.} \end{array}\right. } \end{aligned}$$

Furthermore, as broadcasted signals are binary, the corruption mapping $C(\cdot )$ can be characterized by the conditional probabilities $c_{0|0}$ and $c_{0|1},$ where $c_{i|j} = \mathbb {P}\left\{ C(\pi ) = i | \pi = j \right\} .$

The rest of this work focuses on the case in which the network operator can deduce the corruption function and can estimate the capture risk $p_{\text{b}}.$ Then, the average network miss-detection and false alarm rates for an attack of intensity $p_{\text{b}}$ are defined as

4 $$\begin{aligned} \mathbb {P}\left\{ \text {MD};p_{\text{b}} \right\}& := \mathbb {P}_{1}\left\{ \pi _N(S_N,\varvec{G}_N) = 0 \right\} , \quad \text {and} \end{aligned}$$

5 $$\begin{aligned} \mathbb {P}\left\{ \text {FA};p_{\text{b}} \right\}&:= \mathbb {P}_{0}\left\{ \pi _N(S_N,\varvec{G}_N) = 1 \right\} , \end{aligned}$$

respectively (note that $p_{\text{b}}$ implicitly affects the distribution of $\varvec{G}_N$). The case in which these quantities are unknown can be addressed using the current framework with a min-max analysis, which is left for future studies.

### Problem statement

Our goal is to develop a resilient strategy, in order to provide a reliable estimation of *W* even under a significant number of unidentified Byzantine nodes. Note that in most surveillance applications, miss-detections are more important than false alarms, being difficult to estimate the cost of the worst-case scenario. Therefore, the average network performance is evaluated following the Neyman–Pearson criteria, by setting an allowable false alarm rate $\alpha$ and focusing on reducing the miss-detection rate [72]. By denoting by $\mathcal {P}$ the set of all strategies, we have the following optimization problem:

6 $$\begin{array}{ll}\underset{\{\pi _n\}_{n=1}^\infty \in {\mathcal{P}}}{\text{minimize}} \quad {\mathbb{P}}\left\{ \text {MD};p_{\text{b}} \right\} \\ \text {subject to} \quad {\mathbb{P}}\left\{ \text {FP};p_{\text{b}} \right\} \le \alpha . \end{array}$$

Finding an optimal solution to (6) is a formidable challenge, even for the simple case of networks with start topology and no Byzantine attacks (see [30, 73] and references therein). Therefore, our aim is to develop a sub-optimal strategy that enables resilience, while being suitable for implementation in sensor nodes with limited computational power.

## Social learning as a data aggregation scheme

This section describes our proposed data fusion scheme, and explains its function against topology-aware data falsification attacks. In the sequel, “Data fusion rule” section describes and analyses the data fusion rule, then “Decision statistics” section derives basic properties of its statistics, and finally “An algorithm for computing the social log-likelihood” section presents a practical algorithm for its implementation.

### Data fusion rule

Let us assume that each sensor node is a rational agent that tries to maximizes the profit of an inference within a social network. Rational agents follow *Bayesian strategies*,^6^ which can be elegantly described by the following threshold-based decision rule [72, Chapt. 2]:

7 $$\begin{aligned} \frac{ \mathbb {P}\left\{ W = 1|S_n,\varvec{G}_{n} \right\} }{ \mathbb {P}\left\{ W = 0|S_n,\varvec{G}_{n} \right\} } \mathop {\lessgtr }_{\pi _n=1}^{\pi _n=0} \frac{ u(0,0) - u(1,0) }{ u(1,1) - u(0,1) } . \end{aligned}$$

Above, $u(\pi _n,w)$ is a cost assigned to the decision $\pi _n$ when $W=w,$ which can be engineered in order to match the relevance of miss-detections and false alarms [72].

Let us find a simpler expression for the decision rule (7). Due to the causality constraint (c.f. “System model” section), $\varvec{G}_n$ can only be influenced by $S_1,\dots ,S_{n-1};$ and therefore, it is conditionally independent of $S_n$ given *W*. Using this conditional independence condition, one can find that

8 $$\begin{aligned} \frac{\mathbb {P}\left\{ W = 1|S_n,\varvec{G}_{n} \right\}} {\mathbb {P}\left\{W = 0|S_n,\varvec{G}_{n} \right\}} = e^{\Lambda_S(S_n) + \Lambda_{\varvec{G}_{n}}(\varvec{G}_{n})} \end{aligned},$$

where $\Lambda _S(S_n)$ is the log-likelihood ratio of $S_n$ (c.f. “System model” section) and $\Lambda _{\varvec{G}_{n}}(\varvec{G}_{n})$ is the log-likelihood ratio of $\varvec{G}_{n}.$ Then, using (8) one can re-write (7) as

9 $$\begin{aligned} \Lambda _S(S_n) + \Lambda _{\varvec{G}_n}(\varvec{G}_n) \mathop {\lessgtr }_{\pi _n=1}^{\pi _n=0} \tau _0 , \end{aligned}$$

where $\tau _0 = \log \frac{ \mathbb {P}\left\{ W=0 \right\} }{ \mathbb {P}\left\{ W=1 \right\} } + \log \frac{ u(0, 0) - u(1, 0) }{ u(1, 1) - u(0, 1) }.$ In simple words, (9) states how the *n*-th node should fuse the private and social knowledge: the evidence is provided by the corresponding log-likelihood terms, which are then simply added and then compared against a fixed threshold.^7^

Further understanding of the above decision rule can be attained by studying it from the point of view of communication theory [58]. We first note that the decision is made not over the raw signal $S_n$ but over the “decision signal” $\Lambda _S(S_n).$ Interestingly, the processing done by the function $\Lambda _S(\cdot )$ might serve for dimensionality reduction, as $\Lambda _S(S_n)$ is always a single number even though $S_n$ may be a matrix or a high-dimensional vector. Due to their construction and the underlying assumptions over $S_n$ (c.f. “System model” section), the variables $\Lambda _S(S_n)$ are identically distributed and conditionally independent given $W=w.$ Moreover, by introducing the shorthand notation $\tau _n (\varvec{G}_n) = \tau _0 - \Lambda _{\varvec{G}_n}(\varvec{G}_n),$ one can re-write (9) as

10 $$\begin{aligned} \Lambda _S(S_n) \mathop {\lessgtr }_{\pi _n=1}^{\pi _n=0} \tau _n(\varvec{G}_n) . \end{aligned}$$

Therefore, the decision is made by comparing the decision signal with a decision threshold $\tau _n(\varvec{G}_n),$ which can be efficiently computed using the algorithm proposed in “An algorithm for computing the social log-likelihood” section. Note that this represents a comparison between the sensed data, summarized by $\Lambda _S(S_n),$ and the social information carried by $\tau _n(\varvec{G}_n).$

### Decision statistics

Let us find expressions for the probabilities of the actions of the *n*-th agent, first focusing on the case $n=1.$ Note that

11 $$\begin{aligned} \mathbb {P}_{w}\left\{ \pi _1(S_1) = 0 \right\} = \mathbb {P}_{w}\left\{ \Lambda _S(S_1) < \tau _0 \right\} = F_w^\Lambda (\tau _0) , \end{aligned}$$

where $F_w^\Lambda (\cdot )$ is the c.d.f. of $\Lambda _S$ conditioned on $W=w.$ Then, considering the possibility that the first node could be a Byzantine node, one can show that

12 $$\begin{aligned} \mathbb {P}_{w}\left\{ X_1 =0 \right\}&= p_{\text{b}} \mathbb {P}_{w}\left\{ X_1 = 0|\text { Byzantine} \right\} + (1-p_{\text{b}})\mathbb {P}_{w}\left\{ X_1=0 | \text {not a Byzantine} \right\} \nonumber \\&= p_{\text{b}}( c_{0|0} F_w^\Lambda (\tau _0) + c_{0|1} [ 1 - F_w^\Lambda (\tau _0) ] ) + (1-p_{\text{b}}) F_w^\Lambda (\tau _0) \end{aligned}$$

13 $$\begin{aligned}&=z_0 + z_1 F_w^\Lambda (\tau _0) , \end{aligned}$$

where we are introducing $z_0:= p_{\text{b}}c_{0|1}$ and $z_1:= 1 - p_{\text{b}}(1-c_{0|0} + c_{0|1} )$ as short-hand notation, which are non-negative constants that summarize the strength of the adversary. In particular, when the adversary is powerless then $z_0=0$ and $z_1 = 1,$ and hence $\mathbb {P}_{w}\left\{ \pi _1(S_1)=0 \right\} = \mathbb {P}_{w}\left\{ X_1=0 \right\}.$

By considering the *n*-th node, one can find that

14 $$\begin{aligned} \mathbb {P}_{w}\left\{ \pi _n(S_n,\varvec{G}_n) = 0 |\varvec{G}_n=\varvec{g}_n \right\}&= \int _\mathcal {S} \mathbb {P}_{w}\left\{ \pi _n(s_n,\varvec{g}_n) = 0 | S_n=s \right\} \mu _w(s) \text {d} s \nonumber \\&= \int _\mathcal {S} \mathbb {1}\left\{ \pi _{n} (\varvec{g}_{n}, s) = 0 \right\} \mu _w(s) \text {d} s \end{aligned}$$

15 $$\begin{aligned}&= \mathbb {P}_{w}\left\{ \Lambda _S(s) < \tau _{n}(\varvec{g}_{n}) \right\} \end{aligned}$$

16 $$\begin{aligned}&= F_w^\Lambda (\tau _{n}(\varvec{g}_{n})) . \end{aligned}$$

The first equality is a consequence of the fact that $S_n$ is conditionally independent of $\varvec{G}_n$ given $W=w,$ while the second equality is a consequence that $X_n$ can be expressed as a deterministic function of $\varvec{G}_{n}$ and $S_n,$ and hence, becomes conditionally independent of *W*. Above, (16) shows that $\tau _n$ is a sufficient statistic for predicting $X_n$ with respect to $\varvec{G}_{n}.$ Note that $F_w^\Lambda (x)$ can be directly computed from the statistics of the distribution of $S_n$ (c.f. Appendix A). Moreover, using (16) and following a similar derivation as in (12), one can conclude that

17 $$\begin{aligned} \mathbb {P}_{w}\left\{ X_{n} = 0 | \varvec{G}_{n}= \varvec{g}_{n} \right\} = z_0 + z_1 F_w^\Lambda (\tau _n(\varvec{g}_n) ) . \end{aligned}$$

Let us now study the statistics of $\varvec{G}_n.$ By using the definition of the transition coefficients $\beta _w^n(\varvec{g}_{n+1}|x_n,\varvec{g}_{n}),$ one can find that

18 $$\begin{aligned} \mathbb {P}_{w}\left\{ \varvec{G}_{n+1}=\varvec{g}_{n+1} \right\} = \sum _{\varvec{g}_{n} \in \mathcal {G}_{n}} \sum _{x_n\in \{0,1\}} \beta _w^n(\varvec{g}_{n+1}|x_{n},\varvec{g}_{n}) \mathbb {P}_{w}\left\{ X_{n} = x_n, \varvec{G}_{n} = \varvec{g}_{n} \right\} . \end{aligned}$$

Note that, using the above derivations, the terms $\mathbb {P}_{w}\left\{ X_{n} = x_n, \varvec{G}_{n} = \varvec{g}_{n} \right\}$ can be further expressed as

19 $$\begin{aligned} \mathbb {P}_{w}\left\{ X_{n} = x_{n}, \varvec{G}_{n}=\varvec{g}_n \right\}&= \mathbb {P}_{w}\left\{ X_n=x_{n}| \varvec{G}_n=\varvec{g}_n \right\} \mathbb {P}_{w}\left\{ \varvec{G}_n=\varvec{g}_n \right\} \end{aligned}$$

20 $$\begin{aligned}&= \lambda ( z_0 + z_1 F_w^\Lambda (\tau _n(\varvec{g}_n)),x_n) \mathbb {P}_{w}\left\{ \varvec{G}_n=\varvec{g}_n \right\} , \end{aligned}$$

where $\lambda (p,x) = x (1-p) + (1-x) p.$ Therefore, a closed form expression can be found for (18) recursively over $\varvec{G}_n.$

### An algorithm for computing the social log-likelihood

The main challenge for implementing (9) as a data processing method in a sensor node is to have an efficient algorithm for computing $\tau _n(\varvec{g}_n).$ Leveraging the above derivations, we develop Algorithm 1 as an iterative procedure for computing $\tau _n.$

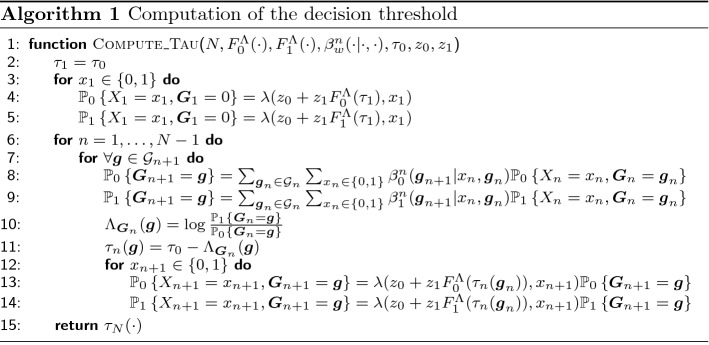



The inputs of Algorithm 1 can be classified into two groups. First, the terms $N,F_0^\Lambda (\cdot ),F_1^\Lambda (\cdot ),\beta _w^n(\cdot |\cdot ,\cdot )$ are properties of the network (position of the node within the decision sequence, sensor statistics and social observability, respectively) that the network operator could measure. On the other hand, $\tau _0,z_0,z_1$ are properties of the adversary profile that depend on the prior statistics of *W*, the rate of compromised nodes $p_{\text{b}}$ and the corruption function defined by $c_{0|0}$ and $c_{0|1}$ (c.f. “The sensor network operator and the adversary” section). In most scenarios, the knowledge of the network controller about these quantities is limited, as attacks are rare and might follow unpredictable patterns. Limited knowledge can still be exploited using e.g. Bayesian estimation techniques [75]. If no knowledge is available for the network controller, then these quantities can be considered free parameters of the strategy that span a range of alternative balances between miss-detections and false positives, i.e. a receiver operating characteristic (ROC) space.

Algorithm 1 initialises from the initial decision threshold $\tau _0,$ and explores all the relevant scenarios iteratively in order to build estimations of the likelihood functions that are required to compute $\tau _N.$ The computation of the terms $\mathbb {P}_{w}\left\{ \varvec{G}_n=\varvec{g} \right\}$ is done following (18), while the ones involving $\mathbb {P}_{w}\left\{ X_n=x_n,\varvec{G}_n=\varvec{g} \right\}$ follow (20). Please note that the algorithm’s complexity scales gracefully for many cases of interest. For the particular case of nodes with memory of length *k* (i.e. $\varvec{G}_n=(X_{n-k-1},\dots ,X_{n-1})$), the complexity of Algorithm 1 is $\mathcal {O}( 2^k N),$ and therefore grows linearly with the size of the network, while being limited in the values of *k* that one can consider. In general, the algorithm complexity scales linearly with *N* as long as the cardinality of $\mathcal {G}_n$ are bounded, or if a significant portion of the terms $\beta _w^n(\varvec{g}_{n+1} | x_n,\varvec{g}_n)$ are zero.

## Information cascade

The term “social learning” refers to the fact that $\pi _n(S_n,\varvec{G}_n)$ becomes a better predictor of *W* as *n* grows; and hence, larger networks tend to develop a more accurate inference. However, as the number of shared signals grows, the corresponding “social pressure” can make nodes to ignore their individual measurements to blindly follow the dominant choice, triggering a cascade of homogeneous behaviour. It is our interest to clarify the role of the social pressure in the decision-making of the agents involved in a social network, as information cascades can introduce severe limitations in the asymptotic performance of social learning [44].

Moreover, an adversary can leverage the information cascade phenomenon. In effect, if the number of Byzantine nodes $N^*$ is large enough then a misleading information cascade can be triggered almost surely, making the learning process to fail. However, if $N^*$ is not enough then the network may undo the pool of wrong opinions and end up triggering a correct cascade.

In the sequel, the effect of information cascades is first studied in individual nodes in “Local information cascades” section. Then, the propagation properties of cascades are explored in “Social information dynamics and global cascades” section.

### Local information cascades

In general, the decision $\pi _n(S_n,\varvec{G}_n)$ is made based on the evidence provided by both $S_n$ and $\varvec{G}_{n}.$ A *local cascade* takes place in the *n*-th agent when the information conveyed by $S_n$ is ignored in the decision-making process due to a dominant influence of $\varvec{G}_n.$ We use the term “local” to emphasize that this event is related to the data fusion of an individual agent. This idea is formalized in the following definition using the notion of conditional mutual information [76], denoted as $I(\cdot ;\cdot |\cdot ).$

#### **Definition 1**

The social information $\varvec{g}_{n} \in \mathcal {G}_n$ generates a *local information cascade* for the *n*-th agent if $I(\pi _n;S_n|\varvec{G}_n = \varvec{g}_n) = 0.$

The above condition summarizes two possibilities: either $\pi _n$ is a deterministic function of $\varvec{G}_n,$ and hence there is no variability in $\pi _n$ once $\varvec{G}_n$ has been determined; or there is still variability (i.e. $\pi _n$ is a stochastic strategy) but it is conditionally independent of $S_n.$ In both cases, the above formulation highlights the fact that the decision $\pi _n$ contains no information coming from $S_n.$ 8

#### **Lemma 1**

*The variables* $\varvec{G}_n \rightarrow \tau _n \rightarrow \pi _n$ *form a Markov Chain* (*i.e.* $\tau _n$ *is a sufficient statistic of* $\varvec{G}_n$ *for predicting the decision* $\pi _n$)

#### *Proof*

Using (16) one can find that

$$\begin{aligned} \mathbb {P}_{w}\left\{ \pi _n|\tau _n,\varvec{G}_n \right\} = \lambda (F_w^\Lambda (\tau _n),X_n) = \mathbb {P}_{w}\left\{ \pi _n|\tau _n \right\} , \end{aligned}$$

and therefore the conditional independency of $\pi _n$ and $\varvec{G}_n$ given $\tau _n$ is clear. $\square$

Let us now introduce the notation $U_s = {{\mathrm{ess\,sup}}}_{s\in \mathcal {S}} \Lambda _S(S_n=s)$ and $L_s = {{\mathrm{ess\,inf}}}_{s\in \mathcal {S}} \Lambda _S(S_n=s)$ for the essential supremum and infimum of $\Lambda _S(S_n),$ being the signals within $\mathcal {S}$ that most strongly support the hypothesis $\{W=1\}$ over $\{W=0\}$ and vice versa.^9^ If one of these quantities diverge, this would imply that there are signals $s\in \mathcal {S}$ that provide overwhelming evidence in favour of one of the competing hypotheses. If both are finite then the agents are said to have *bounded beliefs* [44]. As sensory signals of electronic devices are ultimately processed digitally, the number of different signals that an agent can obtain are finite, and hence their supremum is always finite. Therefore, in the sequel we assume that both $L_s$ and $U_s$ are finite. Using these notions, the following proposition provides a characterization for local information cascades.

#### **Proposition 1**

*The social information* $\varvec{g}_{n} \in \mathcal {G}_n$ *triggers a local information cascade if and only if the agents have bounded beliefs and* $\tau _n(\varvec{g}_{n}) \notin [L_s,U_s]$.

#### *Proof*

Let us assume that the agents have bounded beliefs. From the definition of $F_w^\Lambda,$ which is a cumulative density function, it is clear that if $\tau _n<L_s$ then $F_0^\Lambda (\tau _n) = F_1^\Lambda (\tau _n) = 0,$ while if $\tau _n>U_s$ then $F_0^\Lambda (\tau _n) = F_1^\Lambda (\tau _n) = 1.$ Therefore, if $\tau _n(\varvec{g}_{n}) \notin [L_s,U_s]$ then, according to (16), it determines $\pi _n$ almost surely, making $\pi _n$ and $S_n$ conditionally independent.

To prove the converse by contrapositive, let us assume that $L_s< \tau _n(\varvec{g}_{n}) < U_s.$ Using again (16) and the definition of $U_s$ and $L_s$, one can conclude that this implies that $0< \mathbb {P}_{w}\left\{ \pi _n=0|\varvec{G}_n \right\} < 1$ for both $w\in \{0,1\}.$ This, in turn, implies that the sets $\mathcal {S}^0(\tau ) = \{ s\in \mathcal {S} | \Lambda _S(s) < \tau _n(\varvec{G}_n \}$ and $\mathcal {S}^1(\tau ) = \mathcal {S} - \mathcal {S}^0$ both have positive probability under $\mu _0$ and $\mu _1,$ which in turn implies the existence of conditional interdependency between $\pi _n$ and $S_n$ in this case. $\square$

Intuitively, Proposition 1 shows that a local information cascade happens when the social information goes above the most informative signal that could be sensed. Some consequences of this result are explored in the next section.

### Social information dynamics and global cascades

It is of great interest to predict when a local information cascade could propagate across the network, disrupting the collective behaviour and hence affecting the network performance. The following definition captures how, during a “global information cascade”, the broadcasted signals $X_n$ do not convey information about the corresponding sensor signals anymore.

#### **Definition 2**

The social information $\varvec{g}_n\in \mathcal {G}_n$ triggers a *global information cascade* if $I(X_m;S_m|\varvec{G}_n = \varvec{g}_n) = 0$ holds for all $m\ge n.$

A global information cascade is a succession of local information cascades. As Proposition 1 showed that agents are free from local cascades as long as $\tau _n\in [L_s,U_s],$ one can guess that global cascades are related to the dynamics of $\tau _n.$ These dynamics are determined by the transitions of $\varvec{G}_n,$ which follows the behaviour dictated by the transition coefficients $\beta _w^n(\cdot |\cdot ,\cdot ).$ To further study the social information dynamics, we introduce the following definitions.

#### **Definition 3**

The collection $\{\varvec{G}_n\}_{n=1}^\infty$ is said to have:

1. Strongly consistent transitions if, for any $W=w,$ $\varvec{g}\in \mathcal {G}_n$ and $\varvec{g'}\in \mathcal {G}_{n-1},$ $\beta _w^n( \varvec{g}|1,\varvec{g'} )>0$ implies $\tau _{n}(\varvec{g}) \le \tau _{n-1}(\varvec{g'}),$ while if $\beta _w^n(\varvec{g}|0,\varvec{g'})>0$ implies $\tau _{n}(\varvec{g}) \ge \tau _{n-1}(\varvec{g'}).$
2. Weakly consistent transitions if, for all $\varvec{g}\in \mathcal {G}_n$ and $\varvec{g'}\in \mathcal {G}_{n-1},$ $\tau _{n-1}(\varvec{g'}) \le L_s$ and $\mathbb {P}_{w}\left\{ \varvec{G}_n=g|\varvec{G}_{n-1}=\varvec{g'} \right\} >0$ implies $\tau _{n}(\varvec{g}) \le L_s,$ while $\tau _{n-1}(\varvec{g'}) \ge U_s$ and $\mathbb {P}_{w}\left\{ \varvec{G}_n=\varvec{g}|\varvec{G}_{n-1}=\varvec{g'} \right\} >0$ implies $\tau _{n}(\varvec{g}) \ge U_s.$ 10

Intuitively, strong consistency means that the decision threshold evolves monotonically with respect to the broadcasted signals $X_n.$ Correspondingly, weak consistency implies that $\tau _n$ cannot return to the interval $[L_S,U_S]$ once it goes out of it. Moreover, the adjectives “strong” and “weak” reflect the fact that weak consistency only takes place outside the boundaries of the signal likelihood, while the strong consistency affects all the decision space. Moreover, strongly consistent transitions imply weakly consistent transitions when there are no Byzantine nodes, as shown in the next lemma.^11^

#### **Lemma 2**

*Strongly consistent transitions satisfy the weak consistency condition if* $p_{\text{b}}=0$.

#### *Proof*

See Appendix B. $$\square$$

Next, it is shown that if the evolution of $\varvec{G}_n$ becomes deterministic and 1–1 after leaving the interval $[L_s,U_s]$ (henceforth called *weakly invertible transitions*), then it satisfies the weak consistency condition.

#### **Lemma 3**

*Weakly invertible transitions imply weakly consistent transitions*.

#### *Proof*

See Appendix C. $$\square$$

Now we present the main result of this section, which is the characterization of information cascades for the case of social information that follows weakly consistent transitions.

#### **Theorem 1**

*If the social information have weakly consistent transitions, then every local information cascade triggers a global information cascade*.

#### *Proof*

Let us consider $\varvec{g}_0\in \mathcal {G}_n$ such that it produces a local cascade in the *n*-th node. Then, due to Proposition 1, this implies that $\tau _n(\varvec{g})\notin [L_s,U_s]$ almost surely. This, combined with the weak consistency assumption, implies that $\tau _{n+1}(\varvec{G}_{n+1})\notin [L_s,U_s]$ almost surely. A second application of Proposition 1 shows that $\mathbb {P}_{w}\left\{ \pi = 0 | \varvec{G}_{n+1} \right\}$ is equal to 0 o 1. This, in turn, guarantees that $I(\pi _{n+1}:S_{n+1} | \varvec{G}_{n} = \varvec{g}) = 0$ almost surely, showing that the $(n+1)$-th node experiences a local information cascade because of $\varvec{G}_n = \varvec{g}_0.$

A recursive application of the above argument allows one to prove that $I(\pi _{n+m};S_{n+m} | \varvec{G}_{n} = \varvec{g}) = 0$ for all $m\ge 0,$ proving the existence of a global cascade. $\square$

This theorem has a number of important consequences. Firstly, it provides an intuitive geometrical description about the nature of global cascades for networks with weak consistency. One can imagine the evolution of $\tau _n(\varvec{G}_n)$ as function of *n* as a random walk within the interval $[L_s,U_s].$ Because of the weak consistency condition, if the random walk step out of the interval, it will never come back. Moreover, as a consequence of this theorem, the stepping out of $[L_s,U_s]$ is a necessary and sufficient condition to trigger a global information cascade over the network.

Also, note that when $G_n = \varvec{X}^n$ (i.e. each node overhears all previous decision) one can prove that $G_n$ has weakly invertible transitions. Therefore, Theorem 1 is a generalization of Theorem 1 of [58] to the case of a network with Byzantine nodes.

## Proof of concept

This section illustrates the main results obtained in “Social learning as a data aggregation scheme” and “Information cascade” sections in a simple scenario. In the following, the scenario is described in “Scenario description” section, and numerical simulations are discussed in “Discussion” section.

### Scenario description

Let us consider a sensor network that has surveillance duties over a sensitive geographical area. The sensitive area could correspond to a factory, a drinkable water container or a warzone, whose key variables need to be supervised. The task of the sensor network is, through the observation of these variables, to detect the events $\{W=1\}$ and $\{W=0\}$ that correspond to the presence or absence of an attack to the surveilled area, respectively. No knowledge about of the prior distribution of *W* is assumed.

We consider nodes that have been deployed randomly over the sensitive area, and hence their locations follow a Poisson point process (PPP). The ratio of the area of interest that falls within the range of each sensor is denoted by *r*. If attacks occur uniformly over the surveilled area, then *r* is also the probability of an attack taking place under the coverage area of a particular sensor. Note that, due to the limited sensing range, the miss-detection rate of individual nodes is roughly equal to $1-r.$ As *r* is usually a small number ($5\%$ in our simulations), this implies that each node is extremely unreliable without cooperation.

Each node measures its environment using a digital sensor of *m* levels dynamical range (i.e. $S_n\in \{0,1,\dots ,m-1\}$). Under the absence of an attack, the measured signal is assumed to be normally distributed with a particular mean value and variance. For simplicity of the analysis, we assume that when conditioned in $\{W=0\}$ the signal $S_n$ is distributed following a binomial distribution of parameters (*m*, *q*), i.e.

21 $$\begin{aligned} \mathbb {P}_{0}\left\{ S_n=s_n \right\} = \left( {\begin{array}{c}m\\ s_n\end{array}}\right) q^{s_n} (1-q)^{m-s_n} := f(s_n;m,q) \end{aligned}$$

which, due to the central limit theorem, approximates a Gaussian variable when *m* is relatively large. Moreover, it is assumed that the sensor dynamical range is adapted to match the mean value on the lower third of the sensor dynamical range, i.e. $\mathbb {E} \left\{ S_n |W=0 \right\} = m/3.$ This naturally imposes the requirement $q=1/3.$

Following standard statistical approaches, it is further assumed that the sensors observe the environment looking for anomalous events, i.e. when the measurement is larger than the mean value in more than two standard deviations. This may correspond, for example, to when a specific chemical compound trespasses safe concentration values, or when too much movement has been detected over a given time window (see e.g. [79]). Using the fact that $\text {Var}\{S_n\} = mq(1-q),$ this gives a threshold $T = \mathbb {E} \left\{ S_n \right\} + 2 \sqrt{ \text {Var}\{S_n\} } = np + 2\sqrt{nq(1-q)}.$ Therefore, it is assumed that an attack is related to the event of $S_n$ being uniformly distributed in [*T*, *m*]. Therefore, one finds that

22 $$\begin{aligned} \mathbb {P}_{1}\left\{ S_n=s_n \right\} =\; &(1-r) \mathbb {P}_{1}\left\{ S_n=s_n | \text {attack out of range} \right\} + r \mathbb {P}_{1}\left\{ S_n=s_n | \text {attack in range} \right\} \nonumber \\ =\; &(1-r) f(s_n;m,q) + r \frac{H( s_n - T)}{m-T}, \end{aligned}$$

where *H*(*x*) is the discrete Heaviside (step) function given by

23 $$\begin{aligned} H(x) = {\left\{ \begin{array}{ll} 1 \quad & \text {if } \,x\ge 0 \\ 0 \quad &\text {in other case.} \end{array}\right. } \end{aligned}$$

**Fig. 1:**
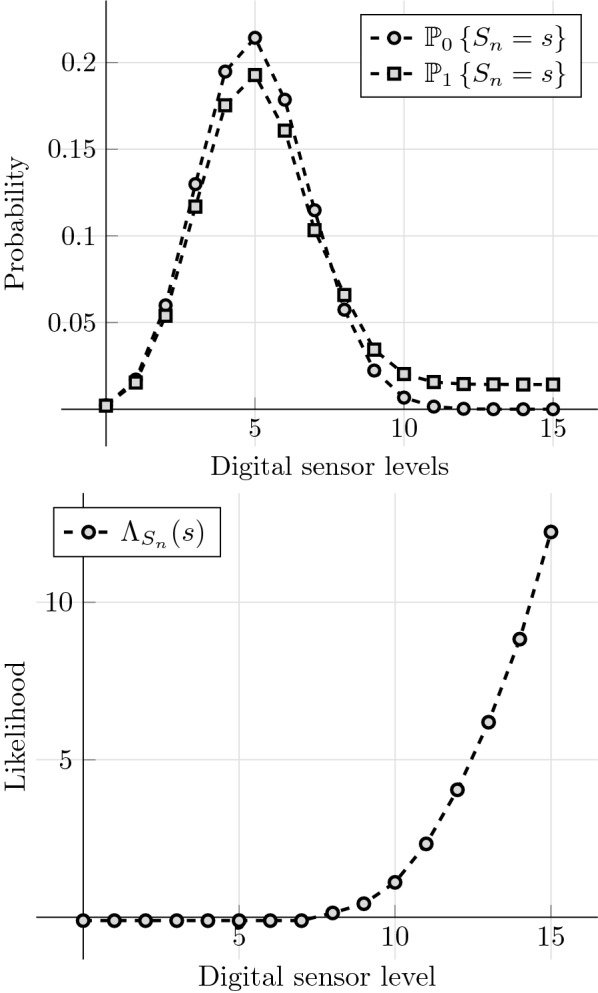
Top: probability distribution for a digital sensor of $m=16$ levels, conditioned on the events $\{W=0\}$ and $\{W=1\}.$ Bottom: Log-likelihood of a digital signal of $m=16$ levels with respect to the variable *W*

In summary, $S_n$ conditioned on $\{W=1\}$ is modelled as a mixture model between a Binomial and a truncated uniform distribution, where the relative weight between them is determined by *r* (c.f. Fig. 1, top). Finally, using (21) and (22), the log-likelihood function of the signal $S_n$ can be determined as (see Fig. 1, bottom)

24 $$\begin{aligned} \Lambda _{S_n}(s_n) = \log \frac{ \mathbb {P}_{1}\left\{ S_n = s_n \right\} }{ \mathbb {P}_{0}\left\{ S_n=s_n \right\} } = \log \left\{ (1-r) + \frac{ r H(s_n - T) }{ (m-T) f(s_n;m,q) } \right\} . \end{aligned}$$

We are interested in studying how a restricted listening period affects the network performance. Restricted listening periods are usually mandatory for energy-limited IoT devices.^12^ For simplicity of the analysis, we focus on scenarios in which a node can overhear the transmissions of all the other nodes, and hence the social information gathered by the *n*-th node is $\varvec{G}_n = (X_{n-k-1},\dots ,X_{n-1})$ if $n > k.$ Here *k* is a design parameter, whose impact on the network performance is studied in the next section.

### Discussion

We analysed the performance of networks of $N=300$ sensor nodes, each of which can monitor $r=5\%$ of the target area. Using the definition given in (4) and (5), combined with (16), miss-detection and false alarm rates are computed as

25 $$\begin{aligned} \mathbb {P}\left\{ \text {MD} \right\} =&\sum _{\varvec{g}\in \mathcal {G}_n} F_1^\Lambda ( \tau _n(\varvec{g})) \mathbb {P}_{1}\left\{ \varvec{G}_n = \varvec{g} \right\} \quad \text {and} \end{aligned}$$

26 $$\begin{aligned} \mathbb {P}\left\{ \text {FA} \right\} =&\sum _{\varvec{g}\in \mathcal {G}_n} (1-F_0^\Lambda ( \tau _n(\varvec{g})) ) \mathbb {P}_{0}\left\{ \varvec{G}_n = \varvec{g} \right\} , \end{aligned}$$

where the terms $\mathbb {P}_{w}\left\{ \varvec{G}_n=\varvec{g} \right\}$ are computed using Algorithm 1 (c.f. “An algorithm for computing the social log-likelihood” section). In order to favour the reduction of miss-detections over false alarms $\tau _0=0$ is chosen, as it is the lowest value that still allows a non-trivial inference process.^13^ We consider an upper bound of $5\%$ over the tolerable false alarm rate.

**Fig. 2:**
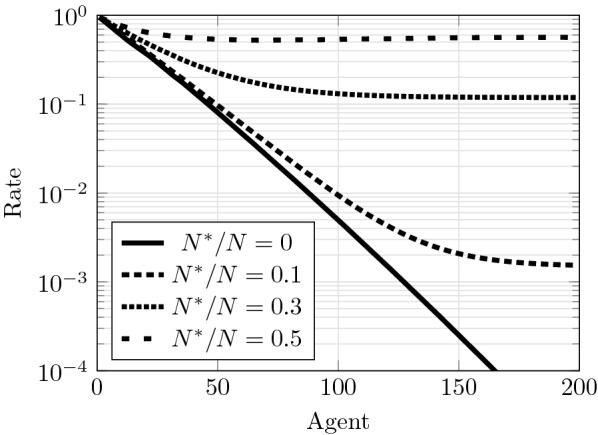
Performance for the inference of each node for various attack intensities, given by the average ratio of Byzantine nodes $N^*/N = p_{\text{b}}.$ Agents overhear the previous $k=4$ broadcasted signals, and use sensors with dynamical range of $n=64$

Simulations demonstrate that the proposed scheme enables strong network resilience in this scenario, allowing the sensor network to maintain a low miss-detection rate even in the presence of a large number of Byzantine nodes (see Fig. 2). Please recall that if a traditional distributed detection scheme based on centralized decision is used, a topology-aware attacker can cause a miss-detection rate of $100\%$ by just compromising the few nodes that perform data aggregation [i.e. the FC(s)]. Figure 2 shows that nodes that individually would have a miss-detection rate of $95\%$ can improve up to around $10\%$ even when $30\%$ of the nodes are under the control of the attacker. Therefore, by making all the nodes to aggregate data, the network can overcome the influence of Byzantine nodes, generating correct inferences even when a significant fraction of nodes have been compromised.

Please note that, for the case of data falsification attack illustrated by Fig. 2, the miss-detection rate improves until the network size reaches $N=500,$ achieving a performance of $\approx 10^{-12}$ (not shown in the Figure). This result has two important implications. First, this confirms the prediction of Theorem 1 that, if the signal log-likelihood is bounded, then information cascades are eventually dominant, hence stopping the learning process of the network (for a more detailed discussion about this issue please c.f. [58]). Secondly, this result stresses a key difference of our approach with respect to the existent literature about information cascades: *even if information cascades become dominant and perfect social learning cannot be achieved, the achieved performance can still be very high, and hence useful in a practical information-processing setup*.

**Fig. 3:**
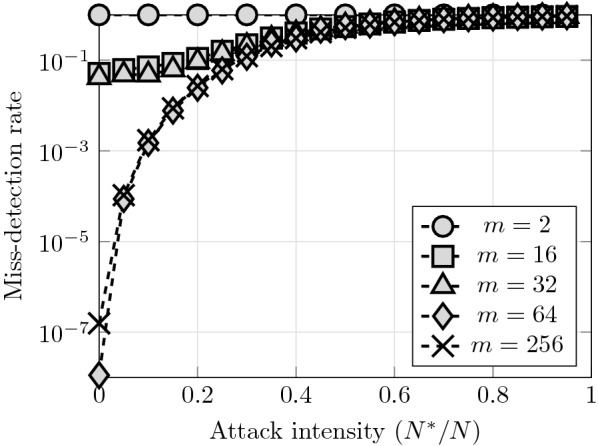
Effect of the sensor dynamical range over the network resilience

The network resilience provided by our scheme is influenced by the sensor dynamical range, *m*, as a higher sensor resolution is likely to provide more discriminative power. Our results show three sharply distinct regimes (see Fig. 3). First, if *m* is too small ($m\le 4$) the network performance is very poor, irrespective of the number of Byzantine nodes. Secondly, if $8\le m \le 32$ the miss-detection rate without Byzantine nodes is approx. $10\%$ (cf. Fig. 3) and is exponentially degraded by the presence of Byzantine nodes. Finally, if $m\ge 64$ then the performance under no Byzantine nodes is very high, and is degraded super-exponentially by the presence of Byzantine nodes. Interestingly, the point at which the miss-detection rate of this regime goes above $10^{-1}$ is $N^*/N=1/3,$ having some resemblance with the well-known 1/3 threshold of the Byzantine generals problem [14]. Also, it is intriguing that variations between 8 and 32 levels in the dynamical range provide practically no performance benefits.

**Fig. 4:**
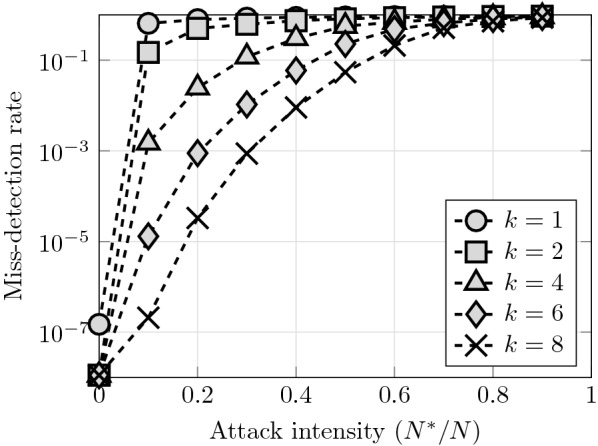
A larger node memory, which allows incorporating more social signals into the inference process, greatly improves the network resilience

Our results also illustrate the effects of the memory size, *k*, showing that larger values of *k* provide great benefits for the network resilience (see Fig. 4). In effect, by performing an optimal Bayesian inference over 8 broadcasted signals the network miss-detection rate remains below $10\%$ up to an attack intensity of $50\%$ of Byzantine nodes. Unfortunately, the computation and storage requirements of Algorithm 1 grow exponentially with *k*, and hence using memories beyond $k=10$ is not practical for resource-limited sensor networks. Overcoming this limitation is an interesting future line of investigation.

## Conclusions

Traditional approaches to data aggregation over information networks are based on a strong division of labour, which discriminates between sensing nodes that merely sense and forward data, and FC that monopolize all the processing and inference capabilities. This generates a single point of failure that is likely to be exploited by smart adversaries, whose interest is the disruption of the network capabilities.

This serious security threat can be overcome by distributing the decision-making process across the network using social learning principles. This approach avoids single points of failure by generating a large number of nodes from where aggregated data can be accessed. In this paper, a social learning data fusion scheme has been proposed, which is suitable to be implemented in sensor networks consisting of devices with limited computational capabilities.

We showed that if the private signals are bounded then each local information cascade triggers a global cascade, extending previous results to the case where an adversary controls a number of Byzantine nodes. This result is highly relevant for sensor networks, as digital sensors are intrinsically bounded, and hence satisfy the assumptions of these results. However, contrasting with the literature, our approach does not focus on the conditions that guarantee perfect asymptotical social learning (i.e. miss-detection and false alarm rates converging to zero), but if their limits are small enough for practical applications. Our results show that this is indeed the case, even when the number of "overheard transmissions is limited.

Moreover, our results suggest that social learning principles can enable significant resilience of an information network against topology-aware data falsification attacks, which can totally disable the detection capabilities of traditional sensor networks. Furthermore, our results illustrate how the network resilience can persist even when the attacker has compromised an important number of nodes.

It is our hope that these results can motivate further explorations on the interface between distributed decision-making, statistical inference and signal processing over technological and social networks and multi-agent systems.

## Appendix A: Properties of $F_w^\Lambda$

For simplicity let us consider the case of real-value signals, i.e. $S_n\in \mathbb {R}.$ In this case, the c.d.f. of the signal likelihood is given by

27 $$\begin{aligned} F_w^\Lambda (y) = \int _{\mathcal {S}^y} \text {d}\mu _w \end{aligned}$$

where $\mathcal {S}^y = \{ x\in \mathbb {R} | \Lambda _s(x) \le y \}.$ If $\Lambda _s$ is an increasing function, then $\mathcal {S}^y=\{x\in \mathbb {R} |x \le \Lambda _s^{-1}(y) \} = (-\infty , \Lambda _s^{-1}(y) ]$ and hence

28 $$\begin{aligned} F_w^\Lambda (y) = \int _{-\infty }^{\Lambda _s^{-1}(y)} \text {d}\mu _w = H_w( \Lambda _s^{-1}(y) ) , \end{aligned}$$

where $H_w(s)$ is the cumulative density function (c.d.f.) of $S_n$ for $W=w.$ For the general case where $\Lambda _s$ is an arbitrary (piece-wise continuous) function, then $\mathcal {S}^y$ can be expressed as the union of intervals. Then $\cup _{j=1}^{\infty } [a_j(y),b_j(y)] = \mathcal {S}^y$ (note that $\Lambda _s(a_j(y))=\Lambda _s(b_k(y))=y$) and hence from (27) is clear that

29 $$\begin{aligned} F_w^\Lambda (y) = \sum _{j=1}^\infty \int _{a_j(y)}^{b_j(y)} \text {d}\mu _w = \sum _{j=1}^\infty \left[ H_w( b_j(y)) - H_w(a_j(y)) \right] . \end{aligned}$$

## Appendix B: Proof of Lemma 2

### *Proof*

Lets assume that the process $\varvec{G}_n$ has strong consistent transitions and consider $\varvec{g'}\in \mathcal {G}_{n-1}$ such that $\tau _{n-1}(\varvec{g'}) \le L_s.$ Note that, under these conditions $F_w^\Lambda (\tau _{n-1}(\varvec{g'})) = 0,$ and hence

30 $$\begin{aligned} \mathbb {P}_{w}\left\{ X_{n-1}=1|\varvec{G}_{n-1}=\varvec{g'} \right\} = 1 - z_0 - z_1 F_w^\Lambda (\tau _{n-1}(\varvec{g'})) = 1 - p_b c_{0|1} = 0 \end{aligned}$$

holds for any $w\in \{0,1\}.$ Moreover, this allows to find that

31 $$\begin{aligned} \mathbb {P}_w\{ \varvec{G}_n=\varvec{g}|\varvec{G}_{n-1}=\varvec{g'}\}&= \sum _{x_n\in \{0,1\}} \beta _w^n(\varvec{g}|x_n,\varvec{g'}) \mathbb {P}_{w}\left\{ X_{n-1}=x_n|\varvec{G}_{n-1}=\varvec{g'} \right\} \nonumber \\&= \beta _w^n(\varvec{g}|1,\varvec{g'}) . \end{aligned}$$

Therefore, due to the strongly consistent transition property, if $\mathbb {P}_{w}\left\{ \varvec{G}_n=\varvec{g}|\varvec{G}_{n-1}=\varvec{g'} \right\} = \beta _w^n(\varvec{g}|1,\varvec{g'}) > 0$ then

32 $$\begin{aligned} L_s \ge \tau _{n-1}(\varvec{g'}) \ge \tau _n(\varvec{g}) , \end{aligned}$$

proving the weak consistent transition property. The proof for the case of $\tau _{n-1}(\varvec{g'}) \ge U_s$ is analogous. $\square$

## Appendix C: Proof of Lemma 3

### *Proof*

Let us consider $\varvec{g}_0\in \mathcal {G}_{n}$ such that $\tau _{n}(\varvec{g}_0) \notin [L_s,U_s].$ Then, due to the weakly invertible evolution, for each $x\in \{0,1\}$ there exists $\varvec{g}(x)\in \mathcal {G}_{n+1}$ such that

33 $$\begin{aligned} \beta _w^n(\varvec{g}|x,\varvec{g}_0) = {\left\{ \begin{array}{ll} 1 \qquad &{}\text {if } \varvec{g}=\varvec{g}(x),\\ 0 \qquad &{}\text {in other case.} \end{array}\right. } \end{aligned}$$

Moreover, note that while the deterministic assumption implies that the event $\{\varvec{G}_{n}=\varvec{g}_0\}$ could be followed by either $\{\varvec{G}_{n+1}=\varvec{g}(0)\}$ or $\{\varvec{G}_{n+1}=\varvec{g}(1)\},$ the 1–1 assumption requires that $\varvec{g}(0) = \varvec{g}(1).$ With this, note that

34 $$\begin{aligned} \Lambda _{\varvec{G}_{n+1}}(\varvec{g}(0)) =&\log \frac{ \mathbb {P}_{1}\left\{ \varvec{G}_{n+1} = \varvec{g}(0) \right\} }{ \mathbb {P}_{0}\left\{ \varvec{G}_{n+1} = \varvec{g}(0) \right\} } \nonumber \\ =&\log \frac{ \sum _{\begin{array}{c} \varvec{g'}\in \mathcal {G}_{n}\\ x \in \{0,1\} \end{array}} \beta _w^n(\varvec{g}(x)|x,\varvec{g'}) \mathbb {P}_{1}\left\{ X_n=x,\varvec{G}_{n}=\varvec{g'} \right\} }{ \sum _{\begin{array}{c} \varvec{g'}\in \mathcal {G}_{n}\\ x\in \{0,1\} \end{array}} \beta _w^n(\varvec{g}(x)|x,\varvec{g'}) \mathbb {P}_{0}\left\{ X_n=x,\varvec{G}_{n}=\varvec{g'} \right\} } \end{aligned}$$

35 $$\begin{aligned} =&\log \frac{ \sum _{x\in \{0,1\}} \mathbb {P}_{1}\left\{ X_n=x|\varvec{G}_n=\varvec{g}_0 \right\} \mathbb {P}_{1}\left\{ \varvec{G}_n = \varvec{g}_0 \right\} }{ \sum _{x\in \{0,1\}} \mathbb {P}_{0}\left\{ X_n=x|\varvec{G}_n=\varvec{g}_0 \right\} \mathbb {P}_{0}\left\{ \varvec{G}_n = \varvec{g}_0 \right\} } \end{aligned}$$

36 $$\begin{aligned} =&\Lambda _{\varvec{G}_{n-1}}(\varvec{g}_0), \end{aligned}$$

Above, (34) is a consequence of $\varvec{g}(0) = \varvec{g}(1),$ while (35) is because of the 1-1 condition over the dynamic. Finally, to justify (36) let us first consider

37 $$\begin{aligned} \mathbb {P}_{w}\left\{ X_n=x|\varvec{G}_n=\varvec{g}_0 \right\} = \lambda ( z_0 + z_1 F_w^\Lambda (\tau _n(\varvec{g}_0)),x) . \end{aligned}$$

Because $\tau _n(\varvec{g}_0) \notin [L_s,U_s]$ then $F_w^\Lambda (\tau _n(\varvec{g}_0))$ is either 0 or 1; in any case it does not depend on *W*. This, in turn means that $\mathbb {P}_{1}\left\{ X_n=x|\varvec{G}_n=\varvec{g}_0 \right\} = \mathbb {P}_{0}\left\{ X_n=x|\varvec{G}_n=\varvec{g}_0 \right\},$ which explains how (36) is obtained.

Please note that (36) shows that, once $\tau _n$ leaves $[L_s,U_s],$ it keeps a constant value. This, in turn, shows that weakly deterministic transitions satisfy the weakly consistency condition. $\square$

## Appendix D: List of symbols

Table2 presents a summary of the notation and symbols used in this work.

**Table 2:** List of symbols and notation

Network properties		
*N*	$\triangleq$	Size of the sensor network
$N^*$	$\triangleq$	Number of Byzantine nodes
$p_{\text{b}}$	$\triangleq$	Probability of a given node being compromised
Sensor and social signals		
$S_n$	$\triangleq$	Signal measured by the *n*-th node
$\mathcal {S}$	$\triangleq$	Set of values that $S_n$ can take
$\mu _w$	$\triangleq$	Distribution of $S_n$ given $W=w$
$\Lambda _S(s)$	$\triangleq$	Log-likelihood of $S_n$ with respect to *W*
$F_w^\Lambda (s)$	$\triangleq$	c.d.f. of $\Lambda _S(s)$ conditioned on $W=w$
$\varvec{G}_n$	$\triangleq$	Social observations of the *n*-th node
$\mathcal {G}_n$	$\triangleq$	Set of values that $\varvec{G}_n$ can take
$\Lambda _{\varvec{G}_n}(\varvec{g})$	$\triangleq$	Log-likelihood of $\varvec{G}_n$ with respect to *W*
$\beta _w^n(\varvec{g} | x_n,\varvec{g'})$	$\triangleq$	Transition probabilities from $\varvec{G}_{n-1}$ to $\varvec{G}_{n}$ given $X_n$ and *W*
Data fusion variables		
*W*	$\triangleq$	Target of the networked inference
$u(\pi _n,w)$	$\triangleq$	Node’s utility function for deciding $\pi _n$ when $W=w$
$\tau _n$	$\triangleq$	Decision threshold used by the *n*-th node
$\pi _n(s,\varvec{g})$	$\triangleq$	Data fusion strategy of the *n*-th node given $S_n$ and $\varvec{G}_n$
$X_n$	$\triangleq$	Signal broadcasted by the *n*-th node
$C(\pi _n), c_{0|0}, c_{0|1}$	$\triangleq$	Corruption function, which links $\pi _n$ and $X_n$
$\mathbb {P}\{\text {MD};p_b\}$	$\triangleq$	Network miss-detection rate
$\mathbb {P}\{\text {FA};p_b\}$	$\triangleq$	Network false alarm rate
Simulation parameters		
*r*	$\triangleq$	Ratio of the area of interest within the sensing range of a single node
*m*	$\triangleq$	Number of quantization levels of a node’s sensor
*k*	$\triangleq$	Node’s memory size

## Footnotes

1 The generalization of our framework and results to vector-valued sensor outputs is straightforward.

2 The conditional independence of sensor signals is satisfied when the sensor noise is due to local causes (e.g. thermal noise), but do not hold when there exist common noise sources (e.g. in the case of distributed acoustic sensors [61]). For works that consider sensor interdependence see [62–66].

3 When $S_n$ takes a finite number of values then $\frac{{\text{d}} \mu _1}{{\text{d}} \mu _0} (s) = \frac{ \mathbb {P}\left\{ S_n=s|W=1 \right\} }{ \mathbb {P}\left\{ S_n=s|W=0 \right\} },$ while if $S_n$ is a continuous random variable with conditional p.d.f. $p(S_n|W=w)$ then $\frac{{\text{d}} \mu _1}{{\text{d}} \mu _0} (s) = \frac{ p(s|W=1) }{ p(s|W=0) }.$

4 Note that the synchronization requirements of this procedure are low, so standard techniques can be used to keep the nodes’ local clocks within the required synchronization constraints (see e.g. [69]).

5 This attack model assumes implicitly that the capture of each node is an independent event. Extensions considering cyber-infection propagation properties are possible (c.f. [71]), being left for future studies.

6 Although Bayesian models are elegant and tractable, they assume agents act always rationally [74] and make strong assumptions on the knowledge agents have about posterior probabilities [49]. However, Bayesian models provide an important benchmark, not necessarily due to their accuracy but because they give an important reference point with which other models can be compared [35].

7 As the prior distribution of *W* is usually unknown, $\tau _0$ is a free parameter of the scheme. Following the discussion in “Problem statement” section, the network operator shall select the lowest value of $\tau _0$ that satisfies the required false alarm rate specified by the Neyman–Pearson criteria.

8 Recall that $S_n$ and $\varvec{G}_n$ are conditionally independent given $W=w$ (c.f. “Data fusion rule” section), and hence there cannot be redundant information about *W* that is conveyed by $S_n$ and also $\varvec{G}_n.$ For a more detailed discussion about redundant information c.f. [77].

9 The essential supremum is the smallest upper bound over $\Lambda _S(S_n)$ that holds almost surely, being the natural measure-theoretic extension of the notion of supremum [78].

10 Note that the condition $\mathbb {P}_{w}\left\{ \varvec{G}_n=\varvec{g}|\varvec{G}_{n-1}=\varvec{g'} \right\} >0$ is equivalent to either $\beta _w^n(\varvec{g},|0, \varvec{g'})$ or $\beta _w^n(\varvec{g},|1, \varvec{g'})$ being strictly positive.

11 It is possible to build examples where weak consistency does not follow from strong consistency when $p_{\text{b}}>0.$

12 It is well known that the wireless radios of small sensor nodes consume a similar amount of energy while transmitting or receiving data, and hence reducing overhearing periods is key for attaining energy efficiency, and hence long network lifetime [60].

13 Simulations showed that if $\tau <0$ then $X_n=1$ for all $n\in \mathbb {N}$ independently of the value of *W*, triggering a premature information cascade.
